# Supplementary figures and images for: Heat shock factor 2 regulates oncogenic gamma-herpesvirus gene expression by remodeling the chromatin at the ORF50 and BZLF1 promoter
Source: PLoS Pathog. 2025 Apr 17;21(4):e1013108. doi: 10.1371/journal.ppat.1013108 (PMC12047821; doi:10.1371/journal.ppat.1013108)

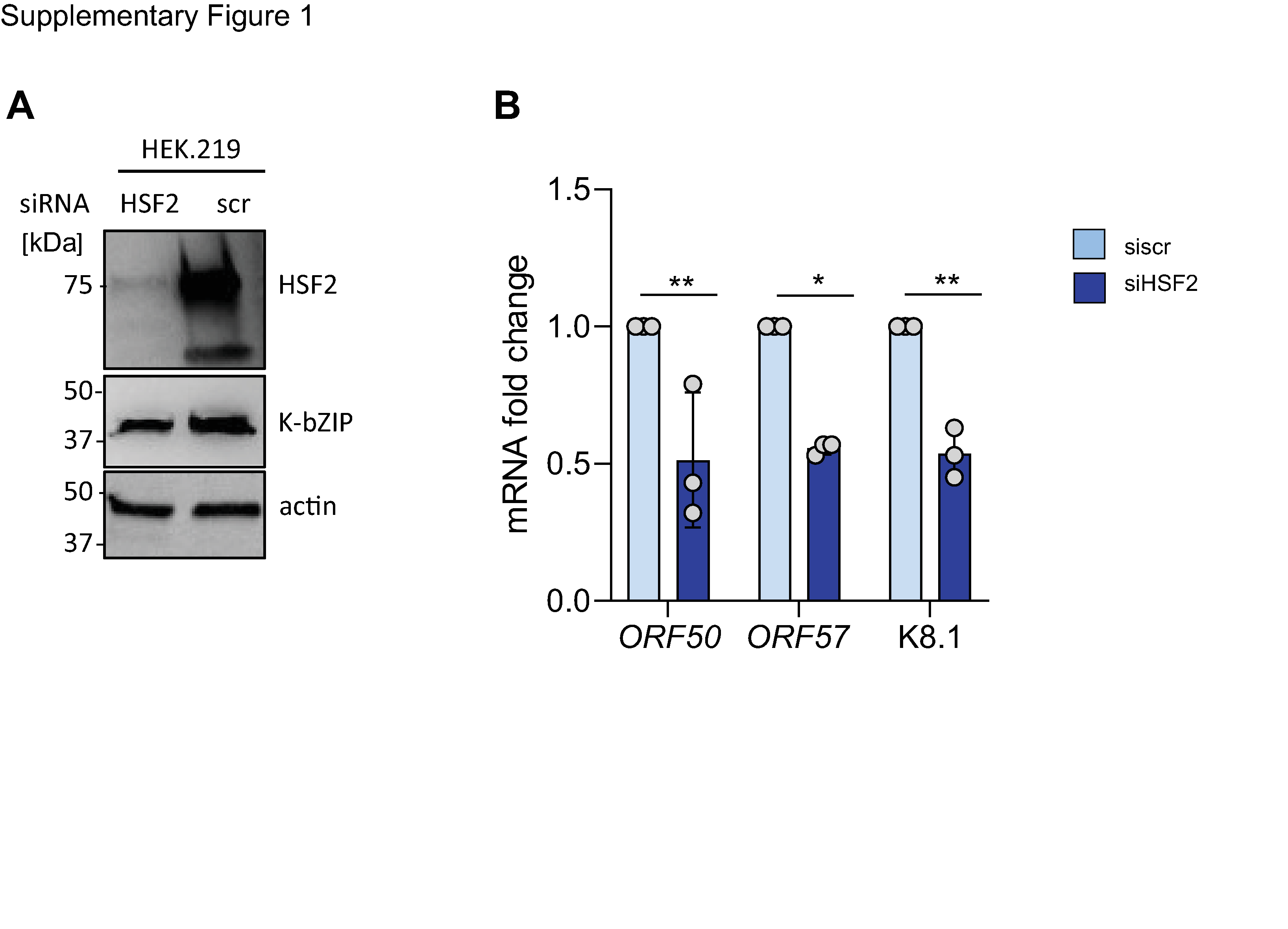

Supplement: S1 Fig — (A) Cells were harvested, and protein levels were analyzed by immunoblot, actin was used as a loading control, molecular weight in kDa is shown on the left side of each blot. (B) RNA was extracted and analyzed for the expression of the indicated viral targets. (TIF) [file ppat.1013108.s001.tif]

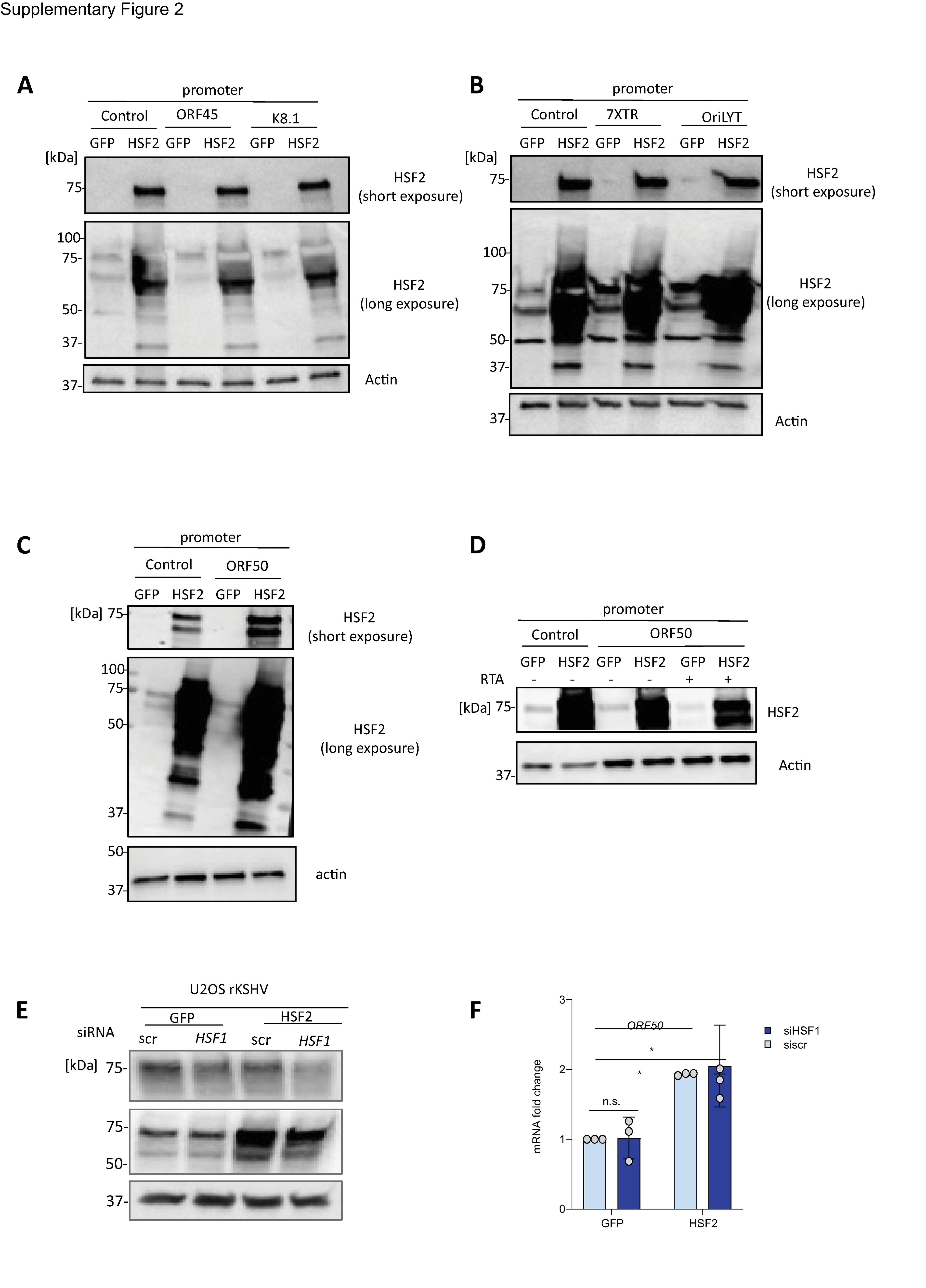

Supplement: S2 Fig — HEK293FT cells were transfected with the indicated plasmids for 24h and after luciferase reporter activity measurement, the protein lysate was analyzed by immunoblot for HSF2 expression, longer membrane exposures are shown to demonstrate endogenous HSF2 expression in GFP transfected cells. Actin was used as loading control. (E) Immunoblot analysis for HSF1 and HSF2 in U2OS rKSHV.219 cells overexpressing GFP or HSF2 and transfected with either control (scr) siRNA or an siRNA targeting HSF1 for 48 hours. Actin was used as a loading control. (F) RTqPCR analysis of cells treated as in (E) for ORF50 gene, actin was used as internal control. (TIF) [file ppat.1013108.s002.tif]

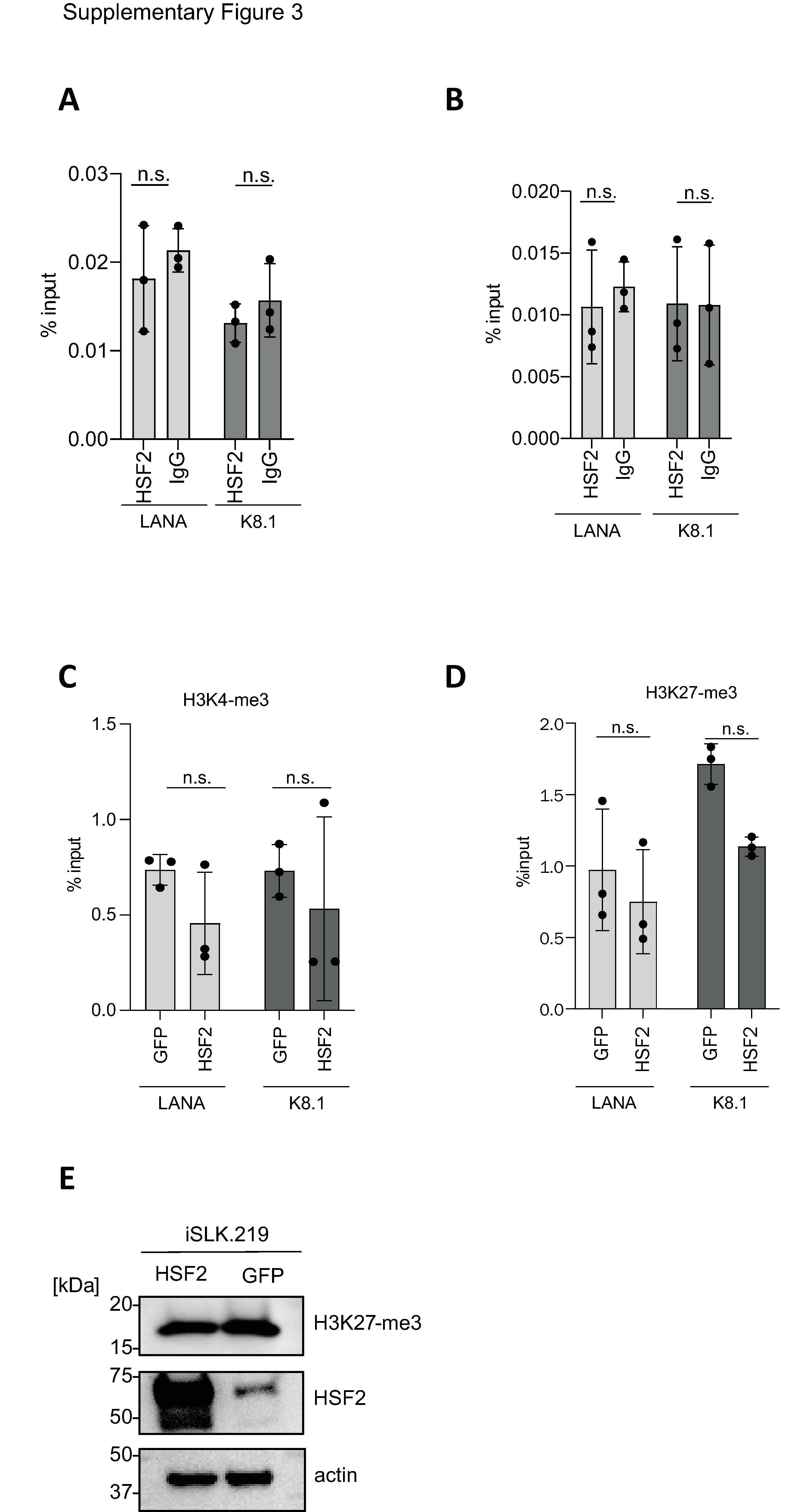

Supplement: S3 Fig — (C and D) ChIP PCR analysis of the indicated histone H3 modification in latent iSLK.219 cells overexpressing HSF2 or GFP. Bars represent the average, and the error bars the SD across three independent experiments, data points are indicated as circles. (E) Immunoblot analysis of iSLK.219 cells overexpressing either HSF2 or GFP control. Actin was used as a loading control. (TIF) [file ppat.1013108.s003.tif]

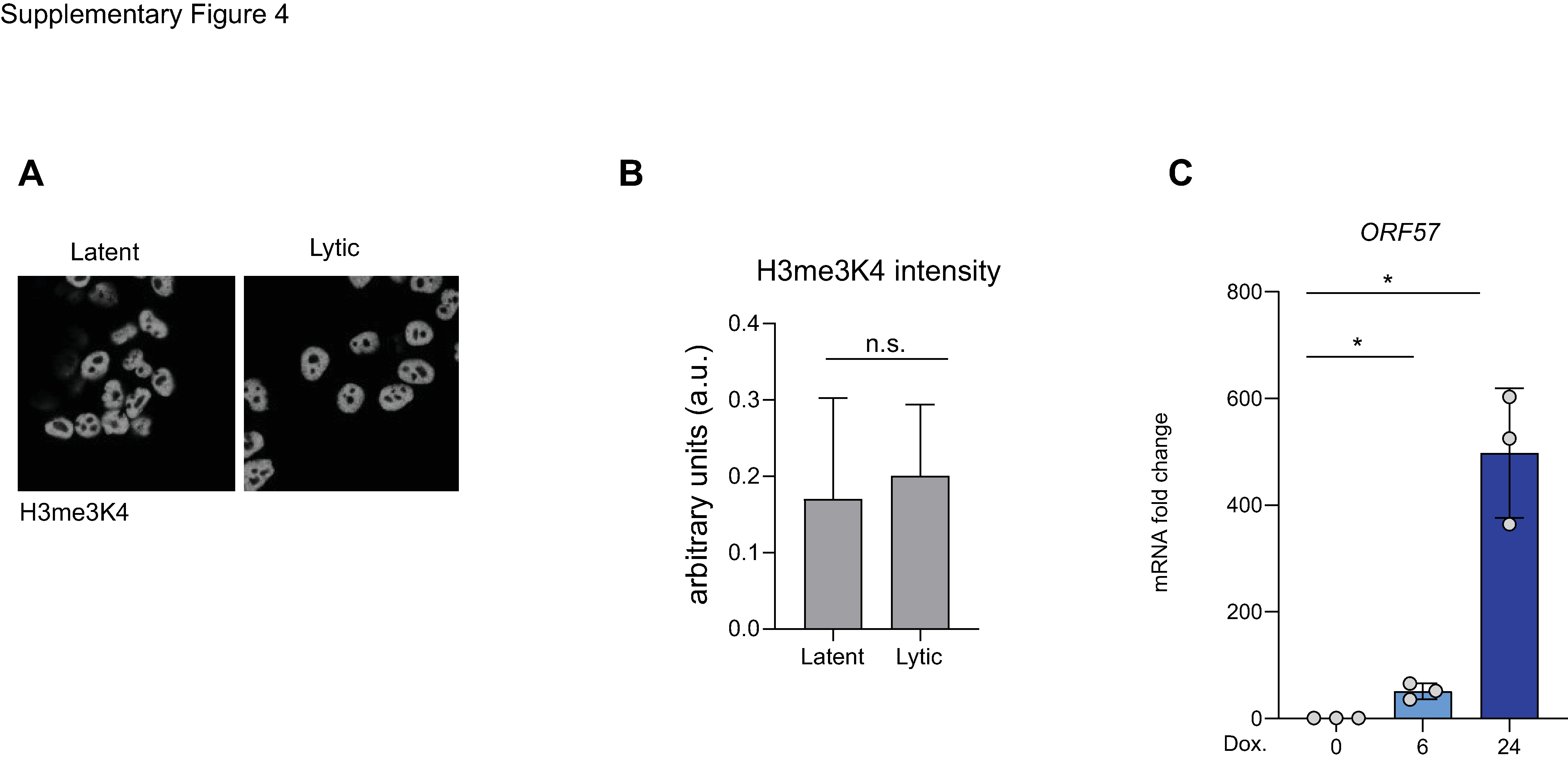

Supplement: S4 Fig — (C) Transcript analysis of ORF57 gene in iSLK.219 cells reactivated with doxycycline (Dox) for the indicated hours. Actin was used as internal control. Bars represent the average and error bars of the SD across three independent experiments, data points are indicated as grey circles. (TIF) [file ppat.1013108.s004.tif]
