# Supplementary material for: Heat shock factor 2 regulates oncogenic gamma-herpesvirus gene expression by remodeling the chromatin at the ORF50 and BZLF1 promoter
Source: PLoS Pathog. 2025 Apr 17;21(4):e1013108. doi: 10.1371/journal.ppat.1013108 (PMC12047821; doi:10.1371/journal.ppat.1013108)
Supplement: Attachment — Submitted filename: Cutrone 25-full-revision point-by-point response.pdf [file ppat.1013108.s005.pdf]

Full Revision

Manuscript number: RC-2024-02798)
Corresponding author(s): Silvia Gramolelli

[Please use this template only if the submitted manuscript should be considered by the affiliate journal as a full revision in response to the points raised by the reviewers.

If you wish to submit a preliminary revision with a revision plan, please use our "Revision Plan" template. **It is important to use the appropriate template to clearly inform the editors of your intentions.**]

1. General Statements [optional]

We thank the Reviewers for their positive feedback and for recognizing the significance and strengths of our study. Their insightful and constructive comments have helped us to improve our manuscript, and we have addressed all their suggestions.

We have modified the manuscript and figures accordingly.

Here, below each comment, please find our response in blue.

2. Point-by-point description of the revisions

Reviewer #1
Evidence, reproducibility and clarity
Summary
In Cutrone et al., the authors discover that the cellular transcription factor Heat shock factor 2 (HSF2) positively regulates the gene expression of two human oncogenic gamma-herpesviruses, Kaposi's sarcoma-associated herpesvirus (KSHV) and Epstein-Barr virus (EBV). In both KSHV and EBV-associated tumors the viral genomes of these viruses are predominantly latent, few viral genes are transcribed, and no virus replication occurs. That said, sporadic reactivation of both KSHV and EBV into a full (called lytic) replication cycle is required for viral dissemination within and between hosts and for tumorigenesis. Although many labs have identified cellular factors that govern the switch between viral latent and lytic states, this remains an incompletely understood phenomenon, largely because the 'switch' does not appear to be the same in all cell types or in all circumstances. Here, Cutrone et al. identify another cellular protein that influences the reactivation of these viruses from latent to lytic state, HSF2. They show that HSF2 binds to the promotors of two key viral transcription transactivators that govern the lytic transcription cascade: ORF50 (also called RTA) for KSHV and Zebra (also called ZTA or BZLF1) to increase their transcription via decreasing repressive chromatin marks in their promoter regions.

Major Comments

Figure 2 The authors report that HSF2 increased the expression of KSHV lytic transcripts in latently infected iSLK.219 cells. As the authors note, these cells have an extremely restrictive transcription program during latent infection without the leaky lytic gene transcription observed in other KSHV models. Since these transcripts are not normally expressed during latency, how are they detected in any reliable measurable way by RT-qPCR? How is the baseline expression in latency detected, where does this value come from as it should be undetectable? At most, I would expect that the authors could report Cq values as below the threshold of detection for lytic genes at baseline in latency. Then, in the presence of overexpressed HSF2, these genes might become detectable or remain below the threshold in the case of HSF2 silencing.

OUR RESPONSE: In response to this relevant comment, we would like to highlight that, under standard conditions, iSLK.219 cells maintain KSHV in a latent state with minimal spontaneous lytic gene expression. For instance, Myoung and Ganem (2011) reported that in the absence of doxycycline, iSLK.219 cells exhibit spontaneous lytic replication in less than 1% of the latent cell population, with lytic gene expression becoming consistently robust only upon induction with doxycycline (PMID: 21419799). We have amended the text accordingly to clarify this point (lanes 143-144).

In our experiments, we observed cycle threshold (Ct) values of approximately 28–31 for the lytic genes ORF50, K8.1, and ORF57 in non-reactivated iSLK.219 cells (see Data for Reviewers 1). In contrast, non-infected control cells (U2OS) exhibited Ct values around 39–40 for all viral targets tested. This indicates that even under latent conditions, iSLK.219 cells exhibit low but detectable levels of lytic gene transcription.
To distinguish true lytic gene expression from background noise, we set a Ct threshold of 35 (indicated by a dotted line in the graphs below), based on Ct values from non-infected cells. Only signals below this threshold were considered indicative of bona fide lytic gene expression. This approach enabled us to detect subtle variations in lytic transcript levels upon HSF2 overexpression or depletion.

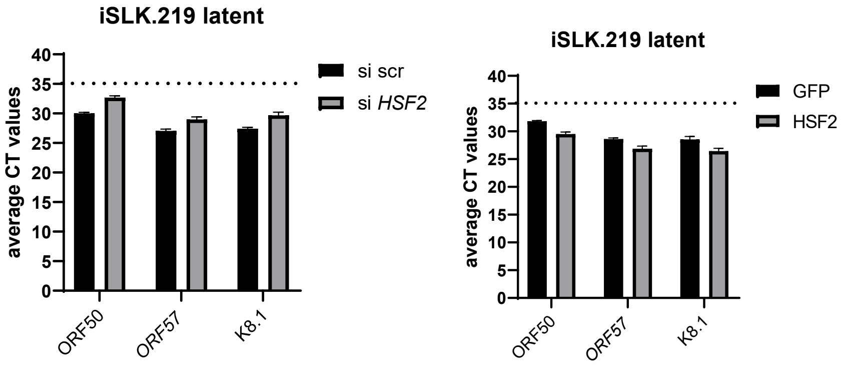

Data for Reviewers 1: Average CT values for the indicated viral targets detected by RTqPCR in latent iSLK.219 treated the indicated siRNAs (left) or transduced with the indicated lentiviruses (right). The dotted line at CT=35 represents the detection threshold. Bars indicate the average, and the error bars the SD across 3 independent experiments.

Fig 2, using this model can you test if virally encoded RTA expression goes up in the presence of HSF2 from undetectable to detectable? Not the Dox-inducible RTA. I presume there is a way to design PCR primers to amplify the viral RTA sequences without picking up the chromosomally integrated form of RTA in these cells. Have one primer bind in the untranslated sequences, perhaps?

OUR RESPONSE: The primers we used to assess ORF50 expression in this study recognize only the endogenous KSHV-encoded ORF50, but not the ectopic doxycycline-induced ORF50, as the forward primer binds across the 5'UTR of the ORF50 gene which is not included in the doxycycline-inducible plasmid, this is specified in the revised result section (lanes 169-170). We have also included this target in latent iSLK.219 where HSF2 was either overexpressed (Figure 2G) or depleted by siRNA (Figure 2H).
Furthermore, we have overexpressed HSF2 also in U2OS rKSHV. This cellular system supports latent KSHV infection and lacks the ectopic Dox.-inducible RTA. In our study we have used this system to complement the finding that HSF1 does not participate to the HSF2-mediated regulation of KSHV infection cycle. As shown in Supplementary Figure 2F, HSF2 overexpression governs ORF50 upregulation also in this system.

Fig 3D is missing an RTA alone control. To show that HSF2 and RTA do not synergize to increase Fluc expression in this assay, please verify that RTA alone does increase the reporter. Without this control it is impossible to know if RTA has the reported effect

OUR RESPONSE: We agree with the Reviewer that this positive control is very important. We have included it in the revised Figure 3D.

Fig 4B. Since all the CHIP is negative in this experiment, can you perform PCR for a cellular promoter that HSF2 should be recruited to and bind under these conditions, need a positive.

OUR RESPONSE: We have included the HSP70 promoter as a cellular control, as HSF2 has been previously shown to bind to it (Östling et al.). The revised Figure 4D now presents these results, demonstrating that HSF2 binds to the HSP70 promoter both in latent cells and after 24 hours of lytic induction

Likewise, when the authors show CHIP for H3K27-me3 they concluded that the decrease in this modification on viral promoters was virus-specific. The specificity control was an immunoblot that shows that HSF2 expression does not change the total level of H3K27-me3. I have never seen this before as a control for histone modifications being unchanged, if this is an established approach please reference. Otherwise, include CHIP for cellular genes that should and should not be repressed under the conditions of the experiment (+/- HSF2) with references.

OUR RESPONSE: In this section of the study, we aimed to determine whether the changes in H3K27me3 levels upon HSF2 overexpression were localized to specific viral regions or broadly distributed. To address this, we performed two key controls: (i) ChIP-qPCR analysis

(Supplementary Figure 3D) confirmed that H3K27me3 levels remained unchanged in other regions of the viral genome, and (ii) an immunoblot to assess the overall levels of H3K27me3. Immunoblotting for this and other histone modifications has been already used to evaluate global histone modification changes following specific treatments or the overexpression/ depletion of a target proteins. Below are recent references as examples (PMID is provided along with information of the figures in every study where the levels of histone modifications were measured by immunoblot):

- Bergwell et al 2024 (PMID: 39209535) (Figure 6B)
- Shokry et al 2024 (PMID: 39482699) (Figures 2B and 2F)
- Naro et al 2024 (PMID: 38413979) (Supplementary Figure S5D)

We have clarified this point and referred to these studies (references number 52-54) in the appropriate revised result section (lanes 258-261)

Fig 5 Why does kBzip protein level diminish with MG132 - is it degraded by autophagy?

OUR RESPONSE: We think there are two possible explanations to the observed reduction of K-bZIP levels 20 h after Dox- induction and upon 3h of MG132 treatment:
1 A functional proteasome is essential for efficient viral replication and for the degradation of cellular proteins that repress lytic reactivation (PMID: 19369342). Inhibiting the proteasome with MG132 may therefore indirectly suppress viral reactivation, by hampering the degradation of cellular repressors of the lytic cycle thus leading to reduced K-bZIP expression.
2 The proteasome plays a critical role in maintaining efficient protein turnover, which is necessary during the early phase of the viral lytic cycle. This has been demonstrated in human cytomegalovirus, where early protein expression (but also several subsequent steps of the viral lytic cycle) was severely impaired upon MG132 treatment (PMID: 18242185). A similar mechanism may contribute, in the context of KSHV infection, to the reduction in K-bZIP levels."

Have you considered that RTA, which functions as an E3 ligase to degrade several cellular proteins in the nucleus during infection, is mediating HSF2 protein decreases? It would be relatively simple to confirm this or rule it out by performing a co-transfection assay of RTA and HSF2. If this were the case, it would provide a nice explanation for why RTA and HSF2 do not synergize to increase Fluc expression. If RTA is not sufficient to induce HSF2 protein decrease, what about RNA silencing of cellular E3 ligases known to elicit turnover of HSF2.

OUR RESPONSE: We thank the Reviewer for prompting us to perform this important experiment. We have used uninfected HEK293 FT cells to ectopically express a MYC-tagged RTA and subsequently assess HSF2 protein levels by immunoblot. Remarkably, we could detect a decrease in HSF2 protein levels upon RTA expression. This result is presented in revised figure 5F and addressed in the results (lanes 311-321) and discussion (lanes 440-445) sections.

The authors present data to show that EBV might also use HSF2 to regulate lytic gene expression. In this case, they report that the EBV master regulator Zebra (also called Zta or

BZRLF1) contains promoter sequences that suggest it could be regulated by HSF2 to elicit viral gene transcription. However, Zta is not the master regulator of most lytic gene transcription for EBV, EBV Rta (also called BRLF1) is. As shown relatively recently, Zta is the first protein expressed after reactivation and is sufficient for induction of lytic origin transcripts BHLF1 and LF3 transcripts, but Rta activates most EBV viral transcripts. Both are required for late gene transcription (since Zta activates the origins) and Zta can cooperate with Rta to enhance transcription of early viral transcripts, but Zta is insufficient to activate early transcription alone (Ali et al 2022. PLoS Pathog. 18(9):e1010886. doi:10.1371/journal.ppat.1010886).
Based on this, if HSF2 induces origin transcripts, this would explain why genome copy number is altered +/- HSF2 as well as viral production and late gene transcription. But the authors are missing some important aspects of EBV transcription regulation and should test EBV Rta in this assay and reconcile the different roles that these two EBV transcription activators have been shown to have.

OUR RESPONSE: Thank you very much for pointing out this important aspect of EBV reactivation mechanism. We have now amended the introduction and cited the indicated work (ref 15) (lanes 62-69). Additionally, we have also tested whether EBV RTA would have an HSF2 consensus binding site on its promoter, however the Jaspar database tool did not find any putative HSF2 binding element. This is now described in the result section (lanes 353-362)

Minor comments

Fig 4D is mislabeled I think this CHIP is for H3K4me3. Fig 4B and 4C need labels on the top of the graphs - latent and lytic.

OUR RESPONSE: This has been corrected and the titles added on the indicated panels.

Fig 6A and 6B - I am pretty sure that the dark blue bars in 6A should be siHSF2 and the light blue should be siscr and in 6B the dark blue should be HSF2 KO and the light blue should be WT. It does not make sense with the text any other way. This is a minor error but caused much confusion for me!

OUR RESPONSE: We apologize for this mistake, and the confusion it has created. The bars are now correctly colored.

Please expand on the regulation of EBV transcription by Rta and Zta to include more recent literature.

OUR RESPONSE: We have implemented this suggestion as described above

Please better highlight how novel this is in the heat shock response field. Is HSF2 working alone to activate a promoter quite rare?

OUR RESPONSE: We have explained in the introduction (lanes 79-93) and discussion (lanes 390-395) that HSF2 can regulate gene expression either in concert with HSF1 or independently. Furthermore, as studies on HSF2 too are more limited in number compared to the research on HSF1, our knowledge on different aspects of HSF2-mediated transcriptional regulation remains limited.

How does this contribute to cell biology understanding of heat shock?

OUR RESPONSE: As explained in the discussion (lanes 421-432 and 462-468), we believe that this work would be mainly informative for the functions of HSF2 in contexts other that acute stress responses where HSF2, although involved, is dispensable (Himanen et al 2022, PMID: 35687139). A growing body or literature has emerged in the recent years showing a role for HSF2 in physiological, developmental and pathological contexts which seem so far independent from the acute stress response *per se*.

How does this contribute to stress induced herpesvirus reactivation?

OUR RESPONSE: We have included a paragraph in the discussion section where we examine the implications of our findings in the broader field of herpesviral reactivation in the context of stress (lanes 462-468)

Significance

Significance

This is a strong manuscript from a new lab that contributes to our understanding of how gammaherpesviruses regulate lytic gene expression in different model systems. Our understanding of the balance between latency and lytic transcription is still incomplete. This is in part due to the variety of models used to study important topic, none of which give the same results, and how to equate these models with the in vivo situation remains unclear. I suspect, based on the advanced level of the discussion, that these authors have some great ideas for how to get at this problem and better understand the 'leaky lytic' gene expression that occurs in vivo. I look forward to their future studies on this important problem. That said, in the current study, the authors have provided another example of a cellular stress induced gene that modifies viral lytic gene expression in different cell types. Alone, this study is an important piece of the puzzle but not a major shift for the KSHV/EBV field.

As noted above, I would like the authors to expand on how the reported activities add to the heat shock factor response field. This is not my area of expertise, and I cannot evaluate the significance of these findings in that area.

This manuscript will be of greatest interest to those who study KSHV and EBV oncogenic viruses.

Reviewer #2

Evidence, reproducibility and clarity

Summary:

An important question in the field of herpesviruses is how lytic reactivation is regulated. Some work has been conducted in the past, but we are far from a complete understanding of this important type of viral regulation. This study demonstrates a distinct mechanism of lytic reactivation that differs from the established role of HSF1 in other viruses. A strength of the work lies in its ability to highlight the importance of HSF2 across two related viral systems (KSHV and EBV). They use both gain-of-function and loss-of-function assays to support their claims in a variety of cell types. Furthermore, they share mechanistic insights into how HSF2 is altering viral gene expression. Taken together, these findings represent a meaningful conceptual advance, suggesting that proteins involved in lytic reactivation may be rapidly degraded once they have exerted their influence, thereby reshaping our understanding of the viral lytic activation process.

Major comments:

It would be helpful to see quantitative levels of HSF2 expression at either the protein or RNA level with the lentivirus overexpression and siRNA repression assays.

OUR RESPONSE: We have quantified the band intensities and reported the relative values normalized to the respective loading controls below each HSF2 blot presented in the figures.

Figure 2: Flow cytometry would be a better assay to measure RFP signal in these assays.

OUR RESPONSE: We agree with the Reviewer that a quantification should be provided for the RFP signal. We have quantified the RFP positive cells (RFP+ cells/DAPI-stained nuclei) for each condition and normalized to the respective controls. This is presented in the revised Figure 2B and 2E)

Fig. 4C: It is surprising that all of the HSF2 binding disappears within 24 hours. The authors note this unexpected finding, but can they show HSF2 is binding to any host promoter at this timepoint? This control would serve to demonstrate that HSF2 binding to chromatin still occurs at this 24-hour timepoint.

OUR RESPONSE: We have now included as cellular control the HSP70 promoter which HSF2 binds in unstimulated conditions (Östling et al). This is shown in the revised Figure 4D.

Fig. 5A-B: This assay need an internal control protein that does not change in the lytic cycle or should be removed.

OUR RESPONSE: We agree with the Reviewer that it is an important control to include. We have therefore included the staining for H3meK4 whose intensity does not change 24h after reactivation compared to latently infected cells. This is presented in Supplementary Figure 4 A,B

The protein levels of HSF2 in the lytically-reactivated cells seems different between Fig. 2D, lane 3, and Fig. 5C, lane 3 at similar timepoints. Please explain.

OUR RESPONSE: The observed difference in band intensity, as noted by the Reviewer, likely results from variations in membrane exposure during imaging. In Figure 5C, HSF2 is expressed endogenously, and we used a longer exposure to more clearly highlight the differences in HSF2 levels across the timepoints. In contrast, Figure 2D aims to demonstrate successful overexpression of HSF2 via lentiviral transduction, where the exposure was intentionally kept shorter to avoid saturation of the bands. Notably, the HSF2 band in lane 1 of panel 2D appears less intense than the band in lane 1 of panel 5C, with both these lanes featuring HSF2 levels in latency. Longer exposures in Figure 2D would show saturated bands in the overexpressing cells, which is why we opted for the exposure shown in the figure

Minor comments:
Fig. 4B-C: A title for these graphs (similar to 4D-E) would help readers.

OUR RESPONSE: We have done as suggested.

Fig. 4D: The graph title should be changed from H3K27-me3 to H3K4-me3.

OUR RESPONSE: This is now corrected.

Fig. 6A-B: Are the legends accidentally swapped?

OUR RESPONSE: This is now corrected.

Reviewer #3
Significance
Research into the regulation of lytic reactivation in herpesviruses is important and not fully understood. Strengths of this work include using multiple cell types, using complementary approaches to manipulate HSF2 expression, using multiple viruses, and sharing mechanistic details. This study extends the knowledge of the importance of proteins that are quickly repressed during the lytic cycle. Many in broad fields of herpes virologists, gene regulation scientists, and chromatin experts would find this work of interest and provides a foundation for further research. Review 3
Evidence, reproducibility and clarity
In the manuscript titled "Heat shock factor 2 positively regulates oncogenic herpesvirus gene expression by remodeling the chromatin landscape" the authors explore the role of HSF2 in cells infected by the oncogenic herpesviruses KSHV and EBV. The authors demonstrate that

HSF2 overexpression enhances KSHV gene expression and virus production in primary endothelial cells as well as viral gene expression in latently and lytically infected kidney epithelial cells. Conversely, HSF2 silencing reduces viral gene expression in both cell lines and the production of infectious virus in KSHV-infected lymphatic endothelial cells (KLECs).

The authors provide mechanistic evidence that HSF2 binds to the ORF50 promoter, leading to a reduction in the repressive histone mark H3K27me3, creating a more permissive chromatin environment for transcription. Interestingly, despite transcriptional upregulation of the HSF2 gene during the lytic cycle of KSHV, the HSF2 protein is degraded, suggesting post-transcriptional regulation. The authors extend their findings to EBV, and show that overexpression of HSF2 similarly promotes viral gene expression, while its silencing reduces the expression of selected viral genes, consistent with the observations in KSHV-infected cells.

The experiments are well executed, and the data support the claims of the paper. Below are comments for the authors consideration

Major

Based on their experiments in iSLK.219 cells, the authors propose a model in which HSF2 binds the promoter of ORF50 in latently infected cells (Fig. 4B) and primes it for activation by remodeling the chromatin environment (Fig. 4E). During the lytic cycle, HSF2 is degraded (Figs. 2 and 5) and released from the ORF50 promoter (Fig. 4C), making it available for active transcription.

1) The experiments supporting this model rely majorly on HSF2 overexpression.

OUR RESPONSE: We would like to highlight that beside the impact of HSF2 overexpression, we have also investigated viral gene expression after we have depleted HSF2 with:

- **i)** siRNA (in KLEC, Fig. 1D,E;F; in iSLK.219 Fig. 2 D. E, F, H, in EBV-positive gastric organoids in Figure 6A)
- **ii)** CrisprKO (in Fig. 6B and C).
- **iii)** In addition, in Figure 4B and 4C and Figure 7B the for the chromatin IP we did not overexpress HSF2 but pulled down endogenous HSF2 using a specific antibody.

Additionally, in the revised manuscript we present also new data on HEK392FT stably infected with rKSHV.219 where HSF2 has been depleted by siRNA in Supplementary Figure1.

To strengthen this hypothesis, the authors should assess the levels of H3K27me3 in the ORF50 promoter during latent infection in the context of HSF2 depletion using siRNA.

Review COMMONS

OUR RESPONSE: We agree that this result would strengthen our hypothesis. We have performed this experiment, and it is included in revised figure 4G

2) In addition to the luciferase reporter experiments shown in Fig. 3, it is important to confirm the upregulation of endogenous ORF50 expression in iSLK.219 cells in the context of HSF2 over expression.

OUR RESPONSE: We have measured endogenous ORF50 expression in latent iSLK.219 both in the context of HSF2 overexpression and HSF2 depletion (by siRNA treatment). This is shown in the revised Figure 2G and 2H.

3) The authors propose a similar mechanism for the BZLF1 promoter of EBV. To validate this hypothesis, they can investigate the occupancy of the BZLF1 promoter by HSF2 and assess the levels of H3K27me3 in this region of the viral genome in HSF2-KO AGS cells (Fig. 6B).

OUR RESPONSE: We have addressed the Reviewer's suggestion and tested by ChIP-PCR the binding of endogenous HSF2 to the BZLF1 promoter. We have found that HSF2 occupies the BZLF1 promoter. This data is presented in Figure 7B.

Regarding the assessment of H3K27me3 levels in HSF2-KO AGS cells, we acknowledge the potential value of this experiment. However, we believe that this additional experiment might be redundant given our current data set which includes:

i) The binding of endogenous HSF2 to the EBV BZLF1 promoter (Fig 7B)
ii) Significantly diminished H3K27me3 levels in HSF2-overexpressing AGS BX1 cells (Fig.7C)
iii) Endogenous HSF2 binding to the ORF50 promoter in latent iSLK.219 (Fig. 4B)
iv) Decreased H3K27-me3 chromatin marks upon HSF2 overexpression at the ORF50 promoter (Fig. 4F)
v) Corresponding H3K27-me3 increase at the KSHV ORF50 promoter when HSF2 was silenced (Fig. 4G).

The authors also suggest that HSF2 modulation of RTA and BZLF1 expression results in leaky lytic viral gene expression in tumors. While the KLEC data supports this hypothesis, exploring other models could provide additional evidence. For example, partial reactivation (~5-10% of cells in latent infection) is reported for PEL-derived cells (e.g., BCBL-1). It would be worthwhile to determine whether endogenous HSF2 levels play a role in promoting viral reactivation in this context. Does silencing HSF2 expression block leaky viral gene expression in BCBL1s?

OUR RESPONSE:

To address the reviewer's insightful comment and explore alternative models of KSHV spontaneous lytic reactivation, we chose to utilize an epithelial cell model specifically,

HEK293FT cells stably infected with rKSHV.219. This model has been previously shown to undergo spontaneous lytic replication in a consistent subset of cultured cells (Myoung and Ganem, 2011, PMID: 2141799). We transiently transfected these cells with siRNA (either control or targeting HSF2) and measured viral gene expression and the levels of the lytic protein K-bZIP. These results are presented in the revised supplementary Figure 1 A,B.

KLEC is currently considered one of the most solid and physiologically relevant models for Kaposi's sarcoma oncogenesis where the virus undergoes spontaneous lytic reactivation. We would like to emphasize that in contrast KSHV infection is predominantly latent in PEL cells, making them the most widely studied model for KSHV latency. This highlight how different is KSHV biology in these two disease models. While only a minute fraction of cells in PEL cultures are lytically infected, in the BCBL-1 PEL model, the subset of lytically infected cells is more prominent; however, this represents an exception rather than the typical mode of KSHV infection in these cells.

Other comments

Expression levels of HSF2 in KLECs and lytic iSLK.219 cells
1) In the immunoblots for HSF2 (Figs. 1A, 1D, 2D), multiple immunoreactive bands are observed. Are these bands isoforms of HSF2? Is this detection pattern reproducible? Does lytic reactivation alter the ratio of these isoforms?

OUR RESPONSE: We thank the Reviewer for raising this very important question. Indeed, HSF2 detection by immunoblot is characterized by distinct patterns of bands that are cell-type and context dependent. Also, during viral infection we often have detected different sets of bands in different conditions. Obtaining these patterns reproducibly was extremely difficult and, strongly dependent on SDS gel acrylamide percentages, running conditions and other experimental factors.

Little is known on whether these different bands are due to isoform variants of HSF2 or to post-translational modifications or to both events combined.

Three isoforms of HSF2 have been predicted (https://www.ncbi.nlm.nih.gov/gene/3298), however the differences in function and expression of these isoforms have not been systematically investigated, to our knowledge. Additionally, in silico, HSF2 is predicted to undergo a wide array of post translational modifications, including SUMOylation, ubiquitylation, phosphorylation and acetylation at multiple amino acid residues. Only a small number of these modifications have been experimentally confirmed and functionally validated (this is reviewed in PMID: 30420555 and 34478606).

Therefore, in this study we decided not to focus on this aspect.

2) In Fig. 1A, endogenous HSF2 levels in KLECs are very low. Is this due to spontaneous lytic reactivation in these cells? How do HSF2 levels in KLECs compare to those in uninfected LECs?

OUR RESPONSE: We have tested whether HSF2 levels change upon infection of primary LEC by immunoblot. We could not observe any important change in HSF2 protein levels. These results are presented below in Data for Reviewers 2.

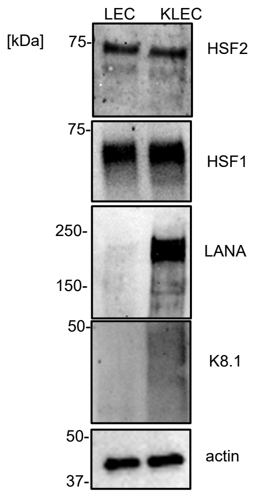

Data for Reviewers 2: primary LEC were infected with rKHSV.219 for 2 weeks or left uninfected. Media was changed every 48-72h. Cells were lysed and analyzed by immunoblot for the indicated cellular and viral proteins. Actin served as loading

3) The striking reduction in HSF2 levels during the lytic cycle is first shown in Fig. 2A, yet this is not mentioned in the results for figure 2 and is addressed later in the manuscript (Fig. 5). Consider moving the section "HSF2 protein is degraded during the lytic cycle" earlier in the manuscript for better flow.

OUR RESPONSE: We appreciate the reviewer's suggestion to reorder the manuscript for better flow. However, we believe that the current structure best maintains the logical progression of our findings. Moving the study of HSF2 kinetics during KSHV reactivation earlier could disrupt the narrative and separate key mechanistic insights from their broader context. Therefore, we respectfully prefer to keep the current order.

In the section, "HSF2 regulates the KSHV ORF50 promoter independently of RTA and HSF1"
1) The authors claim that "HSF2 promotes exclusively and specifically the expression of ORF50 by enhancing its promoter activity." However, while the data show upregulation of ORF50 during HSF2 overexpression, there is no evidence to support the exclusivity of this event. This claim requires more global metrics of viral RNA expression in the presence and absence of HSF2, such as RNA-seq.

OUR RESPONSE: We agree with the Reviewer that with this current data this statement is not supported by concrete evidence. We have removed the indicated sentence accordingly.

2) The authors should clarify that the modulation of lytic genes in latent cells in the absence of RTA occurs specifically in the context of HSF2 overexpression.

Review COMMONS

OUR RESPONSE: We have demonstrated that HSF2 modulates lytic gene expression in latent cells also by siRNA depletion in panel 2H for KSHV upon HSF2 silencing and for EBV in panels 6A-C. However, for the indicated section we agree with this Reviewer: we have performed the luciferase reporter assays and test the dependence on HSF1 in the context of HSF2 overexpression. Therefore, we have specified this in the section title (changed to "ECTOPIC HSF2 regulates the KSHV ORF50 promoter independently of RTA and HSF1") and throughout the text in the indicated results section.

Moreover, as their data suggest, HSF2 overexpression leads to RTA expression, which then triggers the expression of lytic genes. It should be explicit that the observed modulation of lytic genes under HSF2 overexpression might be a consequence of elevated RTA levels.

OUR RESPONSE: we have this sentence in the discussion (lanes 458-461)
"Hence, we propose that HSF2 by promoting ORF50 gene expression, would contribute to the "leakage" of downstream lytic transcripts in latently infected cells within these tumors. In this context, HSF2 could represent a pro-oncogenic host factor supporting the expression of viral lytic oncogenes outside of the traditional productive lytic cycle."

General/Minor Comments
1) Consider quantifying the immunoblots, particularly those supporting changes in the levels of HSF2

OUR RESPONSE: We have done as suggested and quantified the HSF2 blots.

2) Specify in the methods section which virus strain was used to generate KLECs.

OUR RESPONSE: This is now specified.

3) Confirm in figure 6A and B that the siRNA and KO panels are correctly labeled (they seem backwards)

OUR RESPONSE: We apologize for mislabeling, we have now corrected both graphs.

4) In the section "Genetic deletion of HSF2 was also accompanied by diminished intracellular viral genome copies," the reference is currently listed as Fig. 6E but corresponds to Fig. 6D.

OUR RESPONSE: corrected.

5)Fig. 2D is mislabeled as H3K27me3; it should be labeled H3K4me3, as described in the text.

OUR RESPONSE: corrected.

Significance

Review COMMONS

The observations reported in the manuscript support a possible role for HSF2 on the known spontaneous lytic reactivation seen in a subset of cells within tumors caused by oncogenic gamma herpesviruses. Understanding the regulation of viral gene expression in this context will aid in the future development of strategies to treat or control herpesvirus-associated malignancies.

The manuscript is of interest for a specialized virology audience. The strong reliance on overexpression models throughout the manuscript raises important questions about the function of HSF2 in modulating viral gene expression in an endogenous context.

Reviewer #4

Evidence, reproducibility and clarity

Summary

In this paper Cutrone et al. demonstrated that the transcription factor HSF2 can regulate human gamma-herpesviruses KSHV (Kaposi's sarcoma herpesvirus) and EBV (Epstein-Barr virus) lytic cycle. Indeed, the authors found a positive correlation between HSF2 levels and the expression of KSHV and EBV lytic proteins and genes in KSHV-infected cells (including LEC or iSLK.219) and EBV-associated gastric cancer primary cells. ChIP experiments revealed that high levels of HSF2 are associated with lower H3K27-me3 chromatin mark at the early lytic KSHV-ORF50 and EBV-BZLF1 promoters, leading to higher chromatin accessibility. Furthermore, the authors found that HSF2 binds to the ORF50 promoter in an HSF1- and RTA-independent manner, during latency and prior to the initiation of the lytic cycle when HSF2 is degraded. These are interesting results that shed light on the host-mediated regulation of the gamma herpesvirus latent to lytic cycle switch, a process strongly connected to oncogenesis, revealing a novel role for HSF2. However, several points need to be addressed by the authors to justify the conclusions before publication.

Major comments

1. The study is entirely based on in vitro HSF2 overexpression models

OUR RESPONSE: We would like to respectfully highlight that we have investigated viral gene expression also after we have depleted HSF2 with: i) siRNA (in KLEC, Fig. 1D,E;F; in iSLK219 Fig. 2 D. E, F, H, in EBV-positive gastric organoids in Figure 6A) and ii) Crispr KO using sgRNA targeting HSF2 (in Fig. 6B and C). In addition, in Figures 4 B,C,D and 7A the for the chromatin IP we did not overexpress HSF2 but pulled down endogenous HSF2 using a specific antibody and used and IgG antibody as control.

and a validation of the system under stress or stimuli that can physiologically increase HSF2 levels is missing. The manuscript would benefit from the addition of these experiments; alternatively, a discussion on the cellular stresses that may induce HSF2 leading to the reactivation of the lytic cycle of latent viruses should be added.

OUR RESPONSE: We have now considered this important point and included it into the discussion section lanes 462-472.

2. In the Western Blots shown in Fig.1, as well as in the other figures throughout the manuscript, the HSF2 protein is detected as multiple bands that are different in each experiment. This may depend on the different cell lines, since HSF2 exists in different isoforms, or on different gel run methods. Please clarify. The variability across different experiments should be described.

OUR RESPONSE: We thank the Reviewer for raising this very important issue. Indeed, HSF2 detection by immunoblot is characterized by distinct patterns of bands that are cell-type and context dependent. Also, during viral infection we have detected different sets of bands in different conditions. Obtaining these patterns reproducibly was extremely difficult and, strongly dependent experimental factors including, but not restricted to SDS gel acrylamide percentages and running voltage and conditions. Additionally, little is known on whether these different bands are due to different splicing variants of HSF2, different isoforms or to post-translational modifications or to both phenomena combined. Therefore, in this study we decided not to focus on this aspect.

Three isoforms of HSF2 have been predicted (https://www.ncbi.nlm.nih.gov/gene/3298), however the differences in function and expression of these isoforms have never been addressed to our knowledge. Additionally, in silico, HSF2 is predicted to undergo a wide array of post translational modifications, including SUMOylation, ubiquitylation, phosphorylation and acetylation at multiple amino acid residues. Only a very small number of these modifications have been experimentally confirmed and functionally validated in a restricted number of in vitro cellular systems (this is reviewed in PMID: 30420555 and 34478606).

In addition, since it is known that HSF2 exists as a dimer, homotrimer or heterotrimer with HSF1, it would be interesting to characterize the HSF2 conformation under the conditions analysed

OUR RESPONSE: We agree that it would be interesting to address this question. In this regard, very little is known in the field of acute stress response and the developmental processes in which HSF2 has been implicated. For instance, upon the well-studied heat shock response, the composition of the HSFs omo-/heterotrimers binding to the stress-responsive promoters is still unknown and remains an open question.

In our study, we can exclude that the heterotrimeric form with HSF1 would play a role in herpesvirus gene regulation, as HSF1 depletion did not significantly affect HSF2 ability to increase ORF50 gene expression. However, we do not know whether HSF2 binds as a monomer, homodimer, or homotrimer at the viral promoters. Additionally, since binding was detected significantly enriched in a wider region around the HSE, we also considered in the discussion the possibility that HSF2 may be part of a larger protein complex recruited to the viral promoters (lanes 403-409).

3.Fig.2B,E: The authors showed the RFP images as a reporter of lytic phase and GFP images as a reporter of latency. However, GFP seems not to be a good latency reporter since, as stated by the authors on page 4-5, GFP is both constitutively expressed by the rKSHV.219 virus used

to infect iSLK.219 cells but it is also overexpressed by the lentiviral control vector by which 'GFP'-called cells are transduced.

OUR RESPONSE: We presented in these panels the GFP channel merely to demonstrate similar cellular confluency across the different conditions. Regardless, we totally agree that in these conditions GFP was not an ideal choice. Also to convey the request of another Reviewer, we have decided to show nuclei (visualized by DAPI staining) as a measure of cell confluency and RFP as a measure of lytic reactivation.

Minor comments

1.In Fig.1 legend it is stated that 'primary LEC were infected with KSHV for 14 days'. Virus information and infection procedures are missing in the Material and Methods section and should be added.

OUR RESPONSE: we have provided this information in the methods section

Also, it would be interesting to compare HSF2 levels in LEC before and after KSHV infection.

OUR RESPONSE: We have tested whether HSF2 levels change upon infection of primary LEC by immunoblot. We could not observe any important change in HSF2 protein levels. These results are presented below.

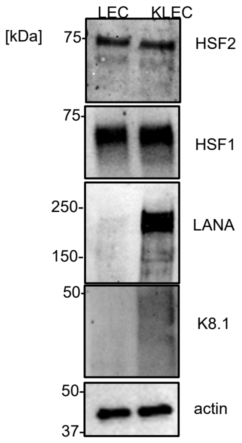

Data for reviewers 2: primary LEC were infected with rKHSV.219 for 2 weeks or left uninfected. Media was changed every 48-72h. Cells were lysed and analyzed by immunoblot for the indicated cellular and viral proteins. Actin served as loading control.

2.Fig.2: As described by the authors iSLK.219 cells used in the experiments contain an ectopic, doxycycline inducible ORF50 gene to induce the lytic cycle. It would be useful to show, in addition to K8.1 and K-bZIP, the ORF50 RNA levels and/or the quantification of virus production both in untreated and Dox-treated cells as a control of lytic cycle induction.

OUR RESPONSE: We present endogenous ORF50 mRNA levels for the iSLK.219 in Fig 2 G and H. In our experimental settings with only 24h of doxycycline induction, no virus can be detected in the supernatant.

3.Fig.3D: It would be useful to add the statistics between HSF2- and GFP-transduced cells.

OUR RESPONSE: Statistics has been added.

4.Fig.3E,F: On page 6, Line 12, the authors stated: 'in line with previous reports documenting lower HSF2 levels in cells devoid of HSF1 (22,23)'. It should be noted that the cited references are focused on the lower HSF2 occupancy on the hsp70 promoter after HSF1 silencing rather than on changes in HSF2 levels after HSF1 silencing; on the other hand, recent studies actually reported that HSF1 regulates HSF2 transcription by directly binding to its promoter under certain conditions. This may explain why, as shown in Fig.3E, HSF1 silencing decrease endogenous HSF2 levels while having no effect on ectopic overexpressed HSF2 that is not under the endogenous HSF2 promoter control. References and discussion should be modified accordingly.

OUR RESPONSE: We have additionally cited the Santopolo et al. 2021 (ref. 44) study where it is reported that HSF1 regulates HSF2 expression during proteasome dysfunction. This is mentioned in the revised results (lane 205) and discussion (lanes 470-471) sections.

In Fig. 3F, is the bar color correct?

OUR RESPONSE: We have corrected the colors of the bars.

5.Fig.4B: ChIP experiments revealed that HSF2 binds to the ORF50 promoter. However, it appears to be no significant enrichment in fragment d containing the HSE. The possibility that the HSF2 binding to ORF50 promoter may be not direct but mediated by other factors should be discussed. In the case of EBV, (Fig.6H) it is interesting the presence of an HSE in the BZLF1 promoter, but this is less relevant without a ChIP analysis for HSF2.

OUR RESPONSE: We have considered the possibility of HSF2 indirect binding to the viral promoters and mentioned it in the discussion sections (lanes 403-409)
We agree with the reviewer that the ChIP analysis of the BZLF1 promoter was a missing piece, hence we have performed this experiment. Results are presented in Figure 7B and show binding of endogenous HSF2 to the BZLF1 promoter in AGS BX1 cells.

6.In Fig.4C the authors demonstrated that HSF2 do not bind to the ORF50 promoter during the lytic phase in both control and HSF2-overexpressing cells. Later in the experiments in Fig.5E, it is shown that MG132 treatment, despite being able to restore HSF2 levels during the lytic cycle preventing its degradation, is not sufficient to induce K-bZIP increase. This suggests that HSF2 cannot bind to the ORF50 promoter during the lytic cycle independently from its protein levels. The possible involvement of other factors that allow HSF2 binding to the ORF50 promoter (see point 5) should be discussed.

OUR RESPONSE: In the experiment shown in Figure 5E (MG132 experiment) we believe the decrease in K-bZIP lytic protein levels is due to the inhibition of the proteasome as previously reported. A functional proteasomal machinery could be needed for efficient herpesviral reactivation and to degrade cellular proteins that have a negative effect on viral lytic cycle (see also above).

7.Fig.4D: Please check panel D legend. Should this be H3K4-me3 instead of H3K27-me3, as described in the text (page 7)?

OUR RESPONSE: Corrected

8.Fig.6A,B: In panels A and B the legend appears to be reversed. Please check.

OUR RESPONSE: Corrected

9.The correlation between HSF2 and the K3H27-m3 levels was analysed only on the KSHV-ORF50 and EBV-BZLF1 promoters; therefore, it would be preferable not to mention a general remodelling of the 'chromatin landscape' in the manuscript title.

OUR RESPONSE: we agree and changed the title accordingly, specifying that the chromatin landscape is remodeled at key viral promoters.

10.Page 8, line 41. Fig. 6E should be Fig. 6D.

OUR RESPONSE: corrected

11.Supplementary Fig.1C: HSF2 long exposure image should be vertically aligned.

12.Some typos (i.e.: Page 29 'CRISP', Page 23 'error bard') and missing punctuation at the end of the sentence (i.e.: Page 22 Line 9) need to be corrected.

OUR RESPONSE: corrected

13.It would be preferable to uniform cell names between results and legends (i.e. Page 22-23: si scr or siscr, Page 29: HSF2 KO or 2KO).

OUR RESPONSE: corrected

Significance
Overall, it is a good quality manuscript. The authors demonstrate the potential ability of HSF2 to regulate the lytic cycle of gamma herpesviruses. However, since this study is entirely based on in vitro HSF2 overexpression models, the major weakness is represented by the lack of a validation of the system under stimuli that can physiologically increase HSF2 levels.

Full Revision

This study would be interesting for the heat shock response field, revealing a new role for HSF2. Furthermore, it also suggests a novel possible molecular process that may affect viral oncogenesis, exploring the host-mediated regulation of the gamma herpesvirus latent to lytic cycle switch.
