# Supplementary material for: Heat shock factor 2 regulates oncogenic gamma-herpesvirus gene expression by remodeling the chromatin at the ORF50 and BZLF1 promoter
Source: PLoS Pathog. 2025 Apr 17;21(4):e1013108. doi: 10.1371/journal.ppat.1013108 (PMC12047821; doi:10.1371/journal.ppat.1013108)
Supplement: Peer review history

# Author comment: Author response to Decision Letter 0

**DOI:** 10.1371/journal.ppat.1013108.r001

31 Mar 2025

Attachment Submitted filename: Cutrone 25-full-revision point-by-point response.pdf

# Editor report: Decision Letter 0

**DOI:** 10.1371/journal.ppat.1013108.r002

**Authors:** Robert F. Kalejta, John Karijolich

7 Apr 2025

Dear Dr. Gramolelli,

We are pleased to inform you that your manuscript 'Heat shock factor 2 regulates oncogenic gamma-herpesvirus gene expression by remodeling the chromatin at the ORF50 and BZLF1 promoter' has been provisionally accepted for publication in PLOS Pathogens.

Before your manuscript can be formally accepted you will need to complete some formatting changes, which you will receive in a follow up email. A member of our team will be in touch with a set of requests.

Please note that your manuscript will not be scheduled for publication until you have made the required changes, so a swift response is appreciated.

IMPORTANT: The editorial review process is now complete. PLOS will only permit corrections to spelling, formatting or significant scientific errors from this point onwards. Requests for major changes, or any which affect the scientific understanding of your work, will cause delays to the publication date of your manuscript.

Should you, your institution's press office or the journal office choose to press release your paper, you will automatically be opted out of early publication. We ask that you notify us now if you or your institution is planning to press release the article. All press must be co-ordinated with PLOS.

Thank you again for supporting Open Access publishing; we are looking forward to publishing your work in PLOS Pathogens.

Best regards,

John Karijolich, Ph.D.

Academic Editor

PLOS Pathogens

Robert Kalejta

Section Editor

PLOS Pathogens

Sumita Bhaduri-McIntosh

Editor-in-Chief

PLOS Pathogens

orcid.org/0000-0003-2946-9497

Michael Malim

Editor-in-Chief

PLOS Pathogens

orcid.org/0000-0002-7699-2064

***********************************************************

Reviewer Comments (if any, and for reference):

# Editor report: Acceptance letter

**DOI:** 10.1371/journal.ppat.1013108.r003

**Authors:** Robert F. Kalejta, John Karijolich

Dear Dr. Gramolelli,

We are delighted to inform you that your manuscript, "Heat shock factor 2 regulates oncogenic gamma-herpesvirus gene expression by remodeling the chromatin at the ORF50 and BZLF1 promoter," has been formally accepted for publication in PLOS Pathogens.

We have now passed your article onto the PLOS Production Department who will complete the rest of the pre-publication process. All authors will receive a confirmation email upon publication.

The corresponding author will soon be receiving a typeset proof for review, to ensure errors have not been introduced during production. Please review the PDF proof of your manuscript carefully, as this is the last chance to correct any scientific or type-setting errors. Please note that major changes, or those which affect the scientific understanding of the work, will likely cause delays to the publication date of your manuscript. Note: Proofs for Front Matter articles (Pearls, Reviews, Opinions, etc...) are generated on a different schedule and may not be made available as quickly.

Soon after your final files are uploaded, the early version of your manuscript, if you opted to have an early version of your article, will be published online. The date of the early version will be your article's publication date. The final article will be published to the same URL, and all versions of the paper will be accessible to readers.

Thank you again for supporting open-access publishing; we are looking forward to publishing your work in PLOS Pathogens.

Best regards,

Sumita Bhaduri-McIntosh

Editor-in-Chief

PLOS Pathogens

orcid.org/0000-0003-2946-9497

Michael Malim

Editor-in-Chief

PLOS Pathogens

orcid.org/0000-0002-7699-2064
